# Inhibition of 6-phosphofructo-2-kinase (PFKFB3) induces autophagy as a survival mechanism

**DOI:** 10.1186/2049-3002-2-2

**Published:** 2014-01-23

**Authors:** Alden C Klarer, Julie O’Neal, Yoannis Imbert-Fernandez, Amy Clem, Steve R Ellis, Jennifer Clark, Brian Clem, Jason Chesney, Sucheta Telang

**Affiliations:** 1 Division of Medical Oncology and Hematology, Department of Medicine, James Graham Brown Cancer Center, University of Louisville, Louisville, KY 40202, USA

**Keywords:** Autophagy, Chemotherapy, Chloroquine, Glycolysis, Reactive oxygen species

## Abstract

**Background:**

Unlike glycolytic enzymes that directly catabolize glucose to pyruvate, the family of 6-phosphofructo-2-kinase/fructose-2,6-bisphosphatases (PFKFBs) control the conversion of fructose-6-phosphate to and from fructose-2,6-bisphosphate, a key regulator of the glycolytic enzyme phosphofructokinase-1 (PFK-1). One family member, PFKFB3, has been shown to be highly expressed and activated in human cancer cells, and derivatives of a PFKFB3 inhibitor, 3-(3-pyridinyl)-1-(4-pyridinyl)-2-propen-1-one (3PO), are currently being developed in clinical trials. However, the effectiveness of drugs such as 3PO that target energetic pathways is limited by survival pathways that can be activated by reduced ATP and nutrient uptake. One such pathway is the process of cellular self-catabolism termed autophagy. We hypothesized that the functional glucose starvation induced by inhibition of PFKFB3 in tumor cells would induce autophagy as a pro-survival mechanism and that inhibitors of autophagy could increase the anti-tumor effects of PFKFB3 inhibitors.

**Results:**

We found that selective inhibition of PFKFB3 with either siRNA transfection or 3PO in HCT-116 colon adenocarcinoma cells caused a marked decrease in glucose uptake simultaneously with an increase in autophagy based on LC3-II and p62 protein expression, acridine orange fluorescence of acidic vacuoles and electron microscopic detection of autophagosomes. The induction of autophagy caused by PFKFB3 inhibition required an increase in reactive oxygen species since N-acetyl-cysteine blocked both the conversion of LC3-I to LC3-II and the increase in acridine orange fluorescence in acidic vesicles after exposure of HCT-116 cells to 3PO. We speculated that the induction of autophagy might protect cells from the pro-apoptotic effects of 3PO and found that agents that disrupt autophagy, including chloroquine, increased 3PO-induced apoptosis as measured by double staining with Annexin V and propidium iodide in both HCT-116 cells and Lewis lung carcinoma (LLC) cells. Chloroquine also increased the anti-growth effect of 3PO against LLCs *in vivo* and resulted in an increase in apoptotic cells within the tumors.

**Conclusions:**

We conclude that PFKFB3 inhibitors suppress glucose uptake, which in turn causes an increase in autophagy. The addition of selective inhibitors of autophagy to 3PO and its more potent derivatives may prove useful as rational combinations for the treatment of cancer.

## Background

Bifunctional 6-phosphofructo-2-kinase/fructose-2,6-bisphosphatases (PFKFBs) regulate glycolytic flux by controlling the steady state concentration of fructose 2,6 bisphosphate (F2,6BP), a potent allosteric regulator of PFK-1 [1]. The PFKFB family consists of four isoforms of which PFKFB3 is of particular interest to the pharmaceutical industry since PFKFB3 mRNA and protein are increased in tumors when compared to normal tissues [2,3]. Although the precise mechanisms for high PFKFB3 expression in human cancers are not fully understood, PFKFB3 mRNA transcription is promoted by HIF-1α [4,5] and by the progesterone receptor [6]. Additionally, loss of the tumor suppressor *PTEN* has recently been found to reduce APC/Cdh1-mediated degradation of PFKFB3 [7] and protein kinase B (AKT) can phosphorylate PFKFB3 resulting in activation [8]. Importantly, deletion of the *Pfkfb3* gene decreases cancer cell glucose metabolism and anchorage-independent growth as soft agar colonies and tumors making this enzyme a promising target for anti-cancer therapy [9] and molecular modeling has allowed for the development of novel small molecule inhibitors that are able to competitively inhibit PFKFB3 enzyme activity.

One such inhibitor, 3-(3-pyridinyl)-1-(4-pyridinyl)-2-propen-1-one (3PO), has been found to suppress glycolytic flux to lactate, decrease glucose uptake and attenuate the proliferation of several human cancer cell lines *in vitro,* including MDA-MB-231 breast adenocarcinoma cells, K-562, HL-60 and Jurkat leukemia cells, HeLa cervical adenocarcinoma cells, and A2058 melanoma cells [10]. Importantly, 3PO has also been found to be selectively cytotoxic to Ras-transformed bronchial epithelial cells relative to untransformed, normal bronchial epithelial cells *in vitro*[10]. Last, 3PO displayed anti-metabolic and anti-tumor effects against Lewis lung carcinoma (LLC), MDA-MB-231 breast and HL-60 leukemic xenograft tumors *in vivo*[10]. Although tumor growth was decreased by treatment with 3PO, it was not completely suppressed, presumably as a result of metabolic resistance mechanisms [10].

Cells in limited nutrient micro-environments, such as those with low amino acid and glucose concentrations, activate the cellular self-digestion process termed autophagy [11-13]. While this process occurs at a basal level within cells playing a complementary role with the proteasome to help clear larger and more abundant material, the induction of autophagy can be triggered by stressful stimuli such as nutrient deprivation. Under these conditions, autophagy is a means by which cells are able to degrade cellular components to provide biosynthetic precursors which can be used for anabolic processes and energy production [14-17]. The induction of autophagy may play an especially critical role in conferring resistance to anti-metabolic drugs since these agents induce states that mimic low nutrient environments. For example, 2-deoxy-glucose has been shown to induce autophagy both *in vitro* and *in vivo* as part of a phase I clinical trial for prostate cancer [18-20].

We postulated that the metabolic stress caused by PFKFB3 inhibition might activate autophagy as a survival pathway, which in turn might confer resistance to 3PO. Chloroquine (CQ), an anti-malarial agent that has been used in humans since the 1940’s, has been shown to inhibit autophagy and potentiate cancer cell death and is now being added to a number of other drugs as a part of several human cancer clinical trials [21-26]. We hypothesized that the combination of the PFKFB3 inhibitor 3PO with the autophagy inhibitor CQ might lead to a significant improvement in the anti-cancer effects of 3PO *in vitro* and that this combination might also increase efficacy of 3PO as an anti-tumor agent *in vivo*. The results of this study demonstrate that PFKFB3 inhibition not only induces autophagy but that CQ can increase the ability of the PFKFB3 inhibitor to cause apoptosis.

## Methods

### Cell culture

Human colorectal carcinoma cells (HCT-116) obtained from the American Type Culture Collection (ATCC, Manassas, VA, USA) were cultured with McCoy’s 5A medium (Gibco, Grand Island, NY, USA) supplemented with 10% calf serum and 50 μg/mL gentamicin. LLC cells obtained from ATCC were cultured in Dulbecco’s Modified Eagle Medium (Gibco) supplemented with 10% calf serum and 50 μg/mL gentamicin. Cells were incubated at 37°C with 5% CO_2_.

### siRNA transfection

HCT-116 cells were plated at 100,000 cells/well in a 6-well dish in 2.5 mL complete medium and, 24 hours after seeding, were transfected with either control siRNA (Stealth Negative Control Medium GC Duplex) or PFKFB3 siRNA (HSS107860 or HSS107862) (all from Invitrogen, Grand Island, NY, USA). For siRNA experiments on LLC cells, cells were transfected with control siRNA (as above) or PFKFB3 siRNA (Silencer Select ID# s100777 Ambion/Invitrogen). ATG5 siRNA was obtained from Invitrogen (ATG5 HSS114103). OptiMEM (Invitrogen) with 1% Lipofectamine RNAiMAX (Invitrogen) was incubated at RT for 5 minutes. siRNA was added to the Lipofectamine mixture and incubated for 20 minutes at room temperature. The mixture was added to a single well of the 6-well plate for a total volume of 3 mL and a final siRNA concentration of 10 nM. Cells were incubated at 37°C for 48 hours before harvest. Samples in which bafilomycin A1 was used were treated with 1 nM bafilomycin A1 (Sigma, St. Louis, MO, USA) for 24 hours prior to harvest.

### Small molecules

3PO was synthesized as previously described [10]; 7,8-dihydroxy-3-(4-hydroxyphenyl) chromen-4-one (YN1) was obtained from Chess (Mannheim, Germany); and CQ, 3-methyladenine, Spautin-1 and bafilomycin A1 were obtained from Sigma.

### Protein extraction

Cells were washed with PBS then lifted in 0.25% trypsin (Gibco) and pelleted by centrifugation. Pellets were lysed in protein lysis buffer (Thermo, Rockford, IL, USA) supplemented with protease and phosphatase inhibitors (Sigma). Samples were homogenized by passing repeatedly through a 28 ½ gauge needle and then incubated on ice for 20 minutes before centrifugation at 2,000 *g* for 5 minutes at 4°C and collection of supernatants. Protein concentration was determined using the bicinchoninic acid assay (Thermo).

### Western blot analyses

Equal amounts of protein were added to loading buffer (BioRad, Hercules, CA, USA) containing 50 μL/mL β-mercaptoethanol and heated to 98°C for 5 minutes and then loaded onto a 4–20% gradient SDS-polyacrylamide gel (BioRad) and run for 60 minutes at 130 volts. The protein was transferred to a nitrocellulose membrane over 1 hour at 400 mA and then blocked in 5% non-fat milk for 1 hour before incubation with primary antibodies. Antibodies against LC3, p62, p-p70S6K, p70S6K, pS6, S6, phospho-AMPK, AMPK, phospho-ULK1, and ULK1 (Cell Signaling, Danvers, MA, USA), PFKFB3 (Proteintech, Chicago, IL, USA), and β-actin (Sigma) were diluted 1:1,000 and incubated overnight at 4°C, with the exception of p62 and β-actin Ab, which were incubated at room temperature for 1 hour. Membranes were washed for 30 minutes in Tris-buffered saline with Tween 20 (TBS-T) (50 mM Tris–HCl, pH 7.4, 150 mM NaCl, 0.1% Tween 20) before addition of secondary antibodies (anti-mouse or anti-rabbit), diluted 1:10,000 in TBS-T (Sigma). ECL Western Blotting Detection Kit (Amersham/GE Pittsburgh, PA, USA) was used to develop membranes. Quantitative densitometry was performed using Image J (NIH).

### F2,6BP assay

Intracellular F2,6BP levels were determined using a method previously described [27]. Briefly, HCT-116 cells were harvested 48 hours after transfection or after treatment with 3PO and centrifuged at 200 *g*. The pellets were resuspended in 50 mM Tris acetate (pH 8.0) and 100 mM NaOH, incubated at 80°C for 5 minutes, and then placed on ice. Extracts were neutralized to pH 7.2 with 1 M acetic acid and 1 M Hepes and then incubated at 25°C for 2 minutes in 50 mM Tris, 2 mM Mg^2+^, 1 mM F6P, 0.15 mM NAD, 10 U/L PPi-dependent PFK-1, 0.45 kU/L aldolase, 5 kU/L triosephosphate isomerase, and 1.7 kU/L glycerol-3-phosphate dehydrogenase. Pyrophosphate (0.5 mM) was added and the rate of change in absorbance (OD = 339 nm) per minute over 5 minutes was determined. A calibration curve using 0.1 to 1 pmol of F2,6BP (Sigma) was used to calculate F2,6BP, which was then normalized to total protein.

### 2-[1-^14^C]-deoxy-D-glucose (2DG)uptake assay

HCT-116 cells were plated at 100,000 cells/well in a 6-well dish. Cells were transfected with either control siRNA or siRNA directed against PFKFB3, or treated with 3PO. Forty eight hours post-transfection or after 3PO treatment, cells were washed with PBS and media was replaced with glucose-free RPMI 1640 (Gibco) for 30 minutes. 2-[1-^14^C]-deoxy-D-glucose (2DG) (Perkin Elmer, Waltham, MA, USA) was added for 30 minutes. Cells were washed three times with ice-cold RPMI 1640 containing no glucose and then lysed with 0.1% SDS. Scintillation counts (counts/min) were measured on a portion of lysate and normalized to protein concentration using the remainder of the lysate. Data are represented as mean ± SD from duplicate samples.

### Acridine orange immunofluorescence

After 48 hours of transfection or after 3PO treatment, HCT-116 cells were washed with PBS and then stained with 0.001 mg/mL acridine orange in PBS for 15 minutes at 37°C. Cells were washed twice with PBS, then harvested for study by microscopy or flow cytometry. For immunofluorescent examination and imaging, cells were viewed using an EVOSfl fluorescent microscope (AMG, Grand Island, NY, USA). Acridine orange was visualized using an overlay of GFP and RFP filters. For flow cytometry, green (510–530 nm) and red (650 nm) fluorescence emission from 10,000 cells illuminated with blue (488 nm) excitation light was measured (BD FACSCalibur, San Jose, CA, USA). FlowJo software (TREE STAR Inc., San Carlos, CA, USA) was used for analysis.

### Electron microscopy

HCT-116 cells were prepared for electron microscopy 48 hours post-transfection or after treatment with 3PO. Cells were washed twice with PBS and fixed in cold glutaraldehyde (3% in 0.1 M cacodylate buffer, pH 7.4) for 30 minutes. Samples were post fixed in OsO_4_ and 100 nm sections were taken and stained with uranyl/lead citrate and viewed using a transmission electron microscope (Phillips CM12). Methodology and identification of autophagic structures was based on established criteria and previous studies [28-30].

### ATP measurement

ATP levels were determined using a bioluminescence assay (Invitrogen) following established protocols from suppliers. Briefly, cells were lysed on cultured plates using 1X passive lysis buffer (Molecular Probes, Carlsbad, CA, USA), snap frozen in liquid nitrogen, then thawed at 37°C and spun at 1,200 *g* for 30 seconds at 4°C to clear the lysates. Lysate was added to a prepared reaction solution containing reaction buffer, DTT, d-luciferin and firefly luciferase, and luminescence was read using a luminometer (TD-20/20, Turner Designs, Sunnyvale, CA, USA). ATP was determined based on a standard curve using 1–500 nM ATP and was calculated relative to protein concentration.

### Reactive oxygen species measurement

2’,7’-dichlorofluorescein diacetate (DCFDA) (1 nM; Invitrogen) was diluted in 1X PBS containing magnesium and calcium (Gibco) and added to washed cells and incubated at 37°C for 30 minutes before being analyzed by flow cytometry (BD FACSCalibur). Data was analyzed using FlowJo software (TREE STAR Inc.). Results were calculated as the average of triplicate samples ± SD.

### Apoptosis assay

Cells were stained with annexin-V labeled with FITC and propidium iodide (PI) following the manufacturer’s protocol (BD Biosciences, San Diego, CA, USA). Briefly, cells were lifted and pelleted by centrifugation at 2,500 rpm for 5 minutes. Cell pellets were washed with 1X PBS and 100,000 cells were pelleted by centrifugation at 2,500 rpm for 5 minutes. Pellets were resuspended in 1X Binding Buffer and annexin-V/FITC and/or PI was added and cells were incubated in the dark at room temperature for 10 minutes. 1X Binding Buffer was added to increase the volume and 10,000 events were counted for each sample using the appropriate filters for FITC and PI detection (BD FACSCalibur). Data was analyzed using FlowJo software (TREE STAR Inc.). Results were calculated as the average of triplicate samples ± SD.

### Tumor model

Twelve-week-old female C57/BL6 mice were injected subcutaneously with 1×10^6^ LLC cells and once tumors reached 150–200 mg, mice were randomized into four groups (n = 6 per group): Group 1, Vehicle (DMSO + PBS); Group 2, Chloroquine (DMSO + 50 mg/kg CQ); Group 3, 3PO (0.07 mg/g 3PO + PBS); Group 4, (0.07 mg/g 3PO + 50 mg/kg CQ). Drug treatments were based on published tumor models [10,31,32]. Mice were given daily intraperitoneal injections with either vehicle or drug and tumors were measured using microcalipers for estimation of tumor volume. At the conclusion of the study, mice were euthanized and tumors were removed. Tumor tissues were fixed in paraformaldehyde and prepared for immunohistochemistry. Animal experiments were carried out in accordance with established practices as described in the National Institutes of Health Guide for Care and Use of Laboratory Animals and were approved by the University of Louisville Institutional Animal Care and Use Committee.

### Immunohistochemistry

Tumors excised after completion of tumor measurements were fixed in paraformaldehyde for 24 hours and then embedded in paraffin, sectioned and stained with an anti-cleaved caspase 3 antibody (1:200, Cell Signaling, Danvers, MA, USA) using standard immunohistochemical methods.

## Results

### Transfection of HCT-116 cells with PFKFB3 siRNA suppresses glucose uptake and increases reactive oxygen species

**Figure 1:**
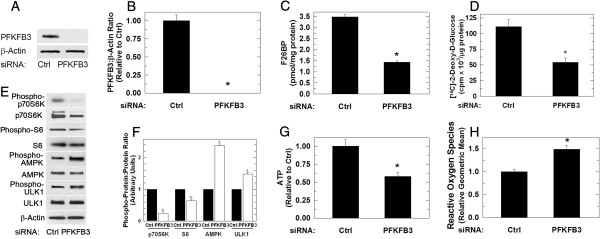
**Transfection of HCT-116 cells with PFKFB3 siRNA inhibits F2,6BP, glucose uptake, ATP and S6K/S6 phosphorylation and simultaneously increases ROS.** HCT-116 cells were transfected with either a control siRNA (ctrl) or 10 nM of a siRNA directed against PFKFB3 (PFKFB3). Total protein was harvested 48 hours post-transfection and protein levels relative to β-actin were determined by Western blotting **(A)**. Densitometry data are presented as the mean fold change ± SD from three experiments **(B)**. F2,6BP levels were determined using an enzyme-coupled assay **(C)**. Glucose uptake was estimated based on the uptake of 2DG **(D)**. After 48 hours of transfection with either a control or a PFKFB3-specific siRNA, protein expression of both phosphorylated and total levels of downstream mTOR targets, p70S6K and ribosomal protein S6 (S6) and of AMPK and ULK1 were measured using Western blotting **(E)**. Quantitative densitometry is reported as phosphorylated protein relative to total protein for p70S6K, S6, AMPK and ULK1 **(F)**. ATP was measured using a bioluminescence assay **(G)** and reactive oxygen species were measured after loading the cells with DCFDA using flow cytometry **(H)**. Data are presented as the mean fold change ± SD of three experiments (*P <0.05).

PFKFB3 expression is high in colon adenocarcinomas and we thus initially transfected HCT-116 colon adenocarcinoma cells with PFKFB3-specific siRNA and confirmed selective suppression of PFKFB3 relative to cells transfected with a control siRNA (Figure 1A, B). Knockdown of PFKFB3 with either PFKFB3-targeted siRNA resulted in a marked reduction in the steady-state concentration of its product, F2,6BP, 48 hours after siRNA transfection (Figure 1C) and decreased cell proliferation (viable cells [× 10^4^/mL]: 24 hours, control, 89 ± 4 and PFKFB3 siRNA, 68.3 ± 3.5; 48 hours, control, 187 ± 8.5 and PFKFB3 siRNA, 78.9 ± 5.4; 72 hours, control, 289.9 ± 8.5 and PFKFB3 siRNA, 85.6 ± 6.3; *P* <0.05 for all time points). Decreased F2,6BP will inhibit PFK-1 activity, which results in an increase of the PFK-1 substrate fructose-6-phosphate (F6P). F6P is in equilibrium with glucose-6-phosphate, an allosteric inhibitor of hexokinase, which itself is required for glucose uptake [33-35]. Accordingly, we suspected that reduced PFK-1 activity caused by PFKFB3 siRNA would suppress glucose uptake as has been observed after deletion of the *Pfkfb3* gene [9]. To measure glucose uptake, 2DG was incubated with cells transfected with the siRNA species - 2DG uptake was reduced by more than 50% after 48 hours in the PFKFB3 siRNA-transfected HCT-116 cells (Figure 1D). These data suggest that PFKFB3 inhibition causes a functional deprivation of glucose similar to that seen in a glucose-poor environment. Importantly, glucose deprivation results in reduction of the mTOR effector ribosomal protein S6 [36] and intracellular ATP [37], and causes an increase in reactive oxygen species (ROS) [38]. The increase in ROS is presumably as a result of decreased mitochondrial membrane potential and depletion of glutathione as previously reported [38,39] or, alternatively, suppression of glycosylation which is also known to result in increased ROS [40]. As has been observed by glucose deprivation, we found that PFKFB3 inhibition reduced intracellular ATP (Figure 1G), increased phosphorylation of both AMPK and ULK1 (Figure 1E, F), inhibited phosphorylation of p70 S6 kinase (p70S6K) and ribosomal protein S6 (S6) (Figure 1E, F), and increased ROS (Figure 1H). Each of these findings is consistent with the concept that PFKFB3 inhibition mimics a glucose-poor environment.

### PFKFB3 knockdown results in activation of autophagy

**Figure 2:**
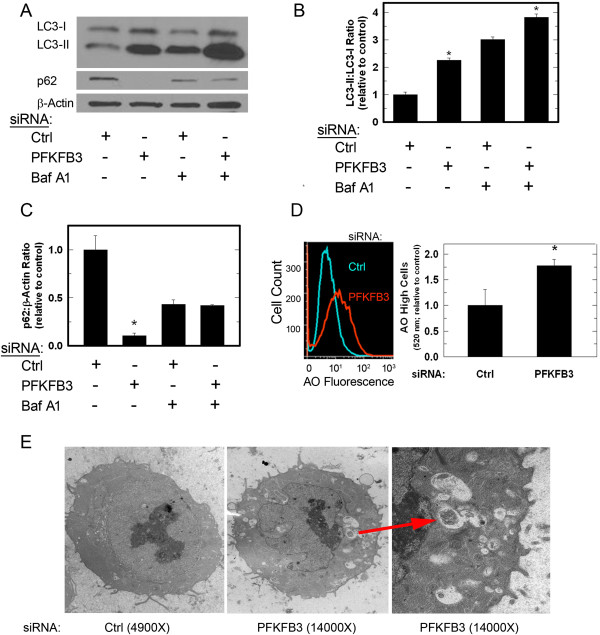
**Transfection of HCT-116 cells with PFKFB3 siRNA stimulates autophagy.** LC3-II and p62 protein levels were determined using Western blotting 48 hours after transfection with either control (ctrl) or a siRNA directed against PFKFB3 (PFKFB3) **(A)**. Treatment with 1 nM bafilomycin A1 (Baf A1) was used to determine if LC3-II levels were a result of increased autophagic flux or impaired degradation **(A)**. Quantitative densitometry was performed to assess relative protein levels **(B, C)**. LC3-II and p62 levels are expressed as the mean fold change ± SD from three experiments relative to LC3-I or β-actin and control. After 48 hours of transfection with either control (ctrl) or PFKFB3-specific siRNA, cells also were stained with acridine orange, observed by fluorescent microscopy and collected by flow cytometry to measure the relative content of acidic compartments **(D)**. Examination of the cells by electron microscopy demonstrated that PFKFB3 siRNA transfection resulted in cells containing intracellular structures consistent with autophagosomes **(E; arrow)**. Data are presented as the mean ± SD from three experiments (*P <0.05).

Glucose starvation induces autophagy and suppression of S6 or ATP and/or an increase in ROS can each result in an increase in autophagy [41,42]. We thus sought to determine if the decrease in glucose uptake due to knockdown of PFKFB3 similarly increased autophagy in HCT-116 cells. Transfection of HCT-116 cells with PFKFB3 siRNA resulted in a significant increase in the microtubule-associated protein 1 light chain 3-II (LC3-II), which is a component of the autophagosomal membrane that is increased during autophagy (Figure 2A, B). Importantly, bafilomycin A1, a vacuolar type H^+^-ATPase that inhibits lysosomal function and is used to block LC3-II degradation, resulted in a further increase in LC3-II, indicating that autophagic flux is increased rather than a block in LC3-II degradation (Figure 2A, B) [43,44]. An additional indicator of autophagy, p62, a ubiquitin-binding scaffold protein that plays a role in the targeting of cargo to autophagosomes where it is degraded, was found to be decreased by PFKFB3 siRNA transfection further supporting an increase in autophagy (Figure 2A, C) [45].

Acridine orange, a cell-permeable fluorescent dye, becomes protonated and trapped in acidic compartments such as lysosomes that are increased in autophagy and, upon excitation (488 nM), emits a red light (650 nM). HCT-116 cells transfected with PFKFB3 siRNA had a significantly higher emission of red light when viewed by fluorescent microscopy (*data not shown*) and PFKFB3 knockdown also resulted in a shift in FL-3 (red) fluorescence by flow cytometry, indicating that the PFKFB3-siRNA transfected cells had a larger quantity of acidic compartments, a characteristic of cells with increased autophagic activity (Figure 2D). Since this is the first demonstration that selective PFKFB3 inhibition causes an induction of autophagy, we also transfected the HCT-116 cells with a second PFKFB3-specific siRNA (see Methods) and confirmed an increase in LC3-II by Western blot analyses and in acridine orange high cells by flow cytometry (Additional file 1: Figure S1).

Another technique commonly used to confirm the process of autophagy is electron microscopy. HCT-116 cells were transfected with PFKFB3 siRNA or a negative control siRNA and, 48 hours post-transfection, were collected and analyzed using a Phillips CM12 transmission electron microscope. An increase in intracellular structures including double-membrane bound vesicles consistent with autophagosomes was observed only in cells transfected with PFKFB3 siRNA (Figure 2E) [46].

### Small molecule inhibition of PFKFB3 decreases glucose uptake and increases ROS

**Figure 3:**
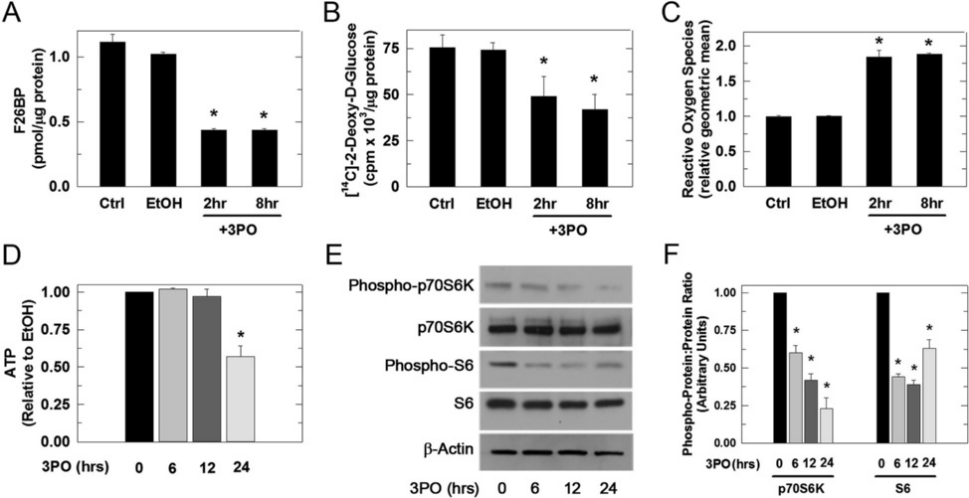
**Small molecule inhibition of PFKFB3 decreases glucose uptake and increases ROS in HCT-116 cells.** HCT-116 cells were treated with 10 μM 3PO and cells were harvested at the indicated time points for the measurement of F2,6BP using an enzyme- coupled assay **(A)**, 2DG uptake **(B)**, ROS by DCFDA staining **(C)**, ATP **(D)**, and phospho-p70S6K, p70S6K, phospho-S6, S6, and β-actin by Western blot **(E)** and densitometry **(F)**. Data are presented as the mean ± SD from three experiments (*P <0.05).

A small molecule designed to target the F6P binding site of the PFKFB3 enzyme, 3PO, has previously been shown to inhibit recombinant PFKFB3 activity and decrease glucose uptake and F2,6BP [10]. To validate this small molecule in our model system, we first examined the effect of 3PO on the proliferation of HCT-116 cells and found a dose dependent inhibition of growth (viable cells [× 10^4^/mL]: 24 hours, control, 21 ± 1.7 and +15 μM 3PO, 4.67 ± 1.2; 48 hours: control, 32.8 ± 0.95 and +15 μM 3PO, 10.2 ± 1.53; 72 hours: control, 39.63 ± 1.7 and +15 μM 3PO, 10.45 ± 1.1; *P* <0.05 for all time points). HCT-116 cells then were treated with either vehicle alone or 10 μM 3PO and F2,6BP levels and glucose uptake were measured. A sharp drop in F2,6BP and glucose uptake was noted after only 2 hours of 3PO exposure (Figure 3A, B). Similar to the PFKFB3 siRNA, we observed an increase in ROS (2 and 8 hours; Figure 3C), and decrease in ATP (24 hours; Figure 3D) and S6 kinase and S6 phosphorylation (6 hours; Figure 3E, F). Taken together with the PFKFB3 siRNA data, these results indicate that selective inhibition of PFKFB3 results in several biochemical changes that occur as a result of the glucose starvation state and that are known to activate autophagy.

### Small molecule inhibition of PFKFB3 induces autophagy

**Figure 4:**
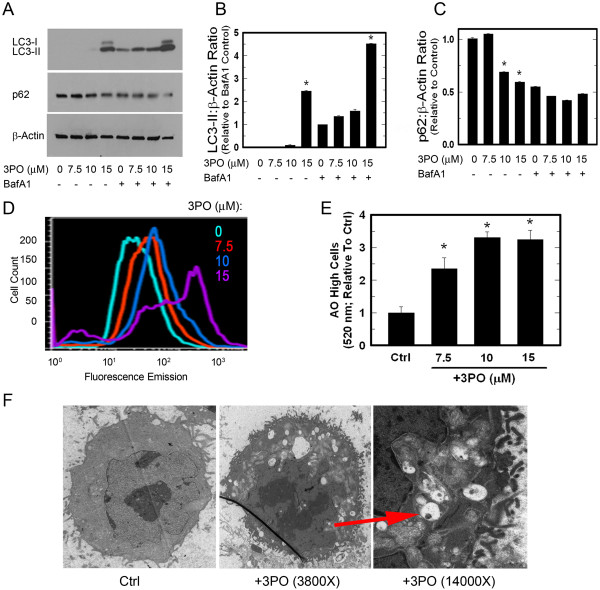
**PFKFB3 inhibition with 3PO stimulates autophagy.** HCT-116 cells were treated with either vehicle, or 7.5, 10, or 15 μM 3PO for 24 hours and LC3-II and p62 expression was measured by Western blot **(A)** and densitometry **(B, C)**. Addition of bafilomycin A1 (Baf A1) was used to determine if the changes in LC3-II were the result of increased synthesis or impaired degradation. LC3-II quantitation is relative to control + bafilomycin due to the absence of a visible band in the control sample. HCT-116 cells were also stained with 1 μg/mL acridine orange for 15 minutes, viewed using a fluorescent microscope, harvested for flow cytometry and gating was used to quantitate the number of cells with a high AO fluorescence and expressed relative to vehicle **(D, E)**. Using electron microscopy, autophagic structures were seen in cells exposed to 3PO **(F; arrow)**.

Like PFKFB3 knockdown, the autophagy marker LC3-II was increased by 3PO and the induction was due to increased synthesis rather than a blockade of protein degradation as indicated by the further increase in LC3-II upon addition of bafilomycin A1 (Figure 4A, B). Also similar to the PFKFB3 siRNA, 3PO resulted in a dose-dependent decrease in p62 (Figure 4A, C) and increased acridine orange immunofluorescence which was visualized by fluorescent microscopy (*data not shown*) and quantified using flow cytometry (Figure 4D, E). Last, after 24 hours of 10 μM 3PO exposure, HCT-116 cells were noted to have numerous intracellular structures consistent with autophagosomes, visualized by electron microscopy (Figure 4F). We confirmed our findings by examining the effects of a second small molecule inhibitor of PFKFB3, YN1, on HCT-116 cells [47]. Cells treated with two concentrations of YN1 or vehicle for 48 hours were counted, F2,6BP levels were measured and were then examined for LC3-II and p62. Similar to 3PO, YN1 decreased viable cell counts, F2,6BP and led to a dose-dependent increase in LC3-II and a decrease in p62 (Additional file 2: Figure S2).

### Activation of autophagy due to PFKFB3 inhibition is reversed with N-acetylcysteine

**Figure 5:**
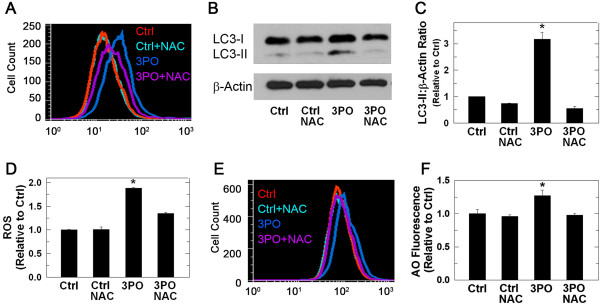
**N-acetylcysteine prevents 3PO-induced ROS and autophagy.** HCT-116 cells were treated with either vehicle or 10 μM 3PO ± 1 mM NAC and harvested after 8 hours of treatment for measurement of DCFDA fluorescence using flow cytometry **(A, D)**. Cell lysates also were prepared and LC3-II levels were determined using immunoblotting **(B)** and densitometry **(C)**. Last, HCT-116 cells were stained with 1 μg/mL acridine orange for 15 minutes and acridine orange fluorescence was determined using flow cytometry **(E, F)**. Data are presented as the mean ± SD from three experiments (*P <0.05).

ROS have been found to stimulate autophagy in part through the mTOR pathway [42]. The observed correlation between oxidative stress and autophagy was further examined using the anti-oxidant N-acetylcysteine (NAC), which can act as a precursor to the antioxidant glutathione. NAC partially blocked the increase in ROS induced by 3PO, as determined by flow cytometric measurement of DCFDA fluorescence (Figure 5A, D). The reduction in ROS following 3PO treatment caused by NAC also blocked the induction of autophagy as measured by loss of LC3-II (Figure 5B, C) and a reduction in acridine orange immunofluorescence (Figure 5E, F). Although these data suggest that the observed increase in autophagy caused by 3PO is dependent on an increase in ROS caused by PFKFB3 inhibition, the high concentration of NAC that was used (1 mM) may have non-specific effects on autophagy as well as on the cytostatic effects of 3PO. Importantly, given the effects of PFKFB3 inhibition on mTOR signaling, AMPK phosphorylation and ROS, we suspect that the induction of autophagy observed after PFKFB3 inhibition is due to multiple indirect and direct mechanisms.

### Pharmacologic inhibition of autophagy in combination with 3PO increases tumor cell death

**Figure 6:**
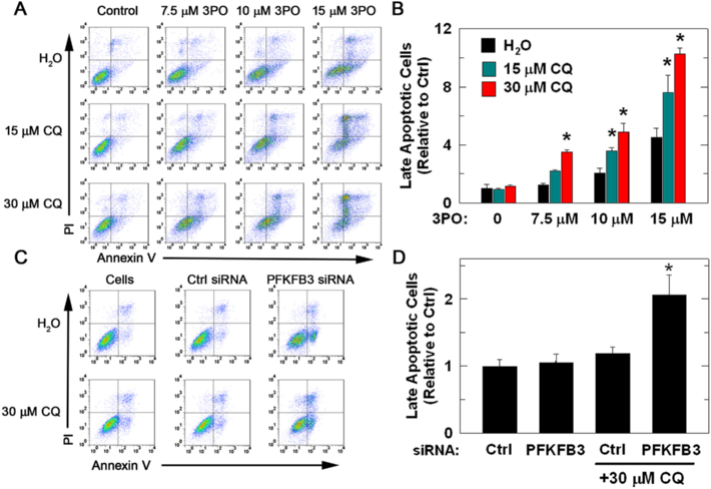
**Chloroquine increases the number of late apoptotic cells caused by exposure to 3PO or PFKFB3 siRNA transfection.** Annexin-V/PI staining was performed in cells treated with vehicle, 3PO, CQ, or the combination of 3PO and CQ for 48 hours. Quantitation of cell staining was performed using flow cytometry **(A)** and the number of cells staining with both annexin-V and PI was quantitated and is expressed as the percentage relative to control ± SD from three experiments **(B)**. HCT-116 cells also were transfected with either a negative control siRNA or with a PFKFB3-specific siRNA ± 30 μM CQ. After 48 hours of transfection, cells were stained with annexin-V and PI and measured using flow cytometry **(C)**. Quantitation of cells staining positive for annexin-V and PI was performed relative to control and is expressed as the mean ± SD from three experiments **(D)** (*P <0.05).

**Figure 7:**
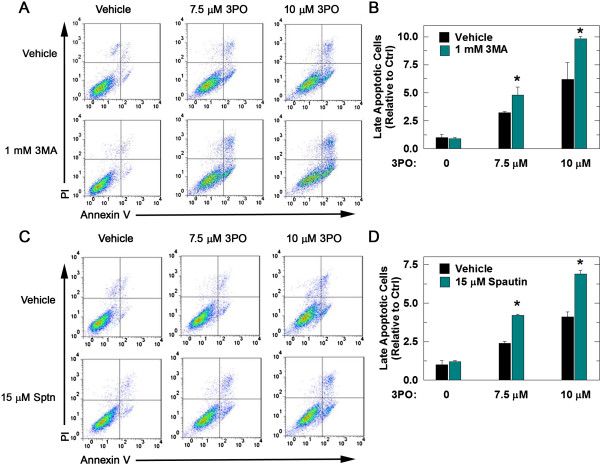
**The autophagy inhibitors, 3-methyladenine and Spautin-1, increase the pro-apoptotic effects of 3PO.** HCT-116 cells were treated with either vehicle or 7.5 μM or 10 μM 3PO ± 1 mM 3-methyladenine (3MA). Forty eight hours after treatment, cells were stained with annexin-V and PI and measured using flow cytometry **(A)**. Cell death was quantitated as the percentage of cells staining positive for annexin-V and PI relative to control ± SD from three experiments **(B)**. HCT-116 cells then were treated with either vehicle, 7.5 or 10 μM 3PO ± 15 μM Spautin-1 and, 48 hours after treatment, cells were stained with annexin-V and PI and collected by flow cytometry **(C)**. The percentage of cells stained positive for both annexin-V and PI was quantitated and is expressed relative to control ± SD from three experiments **(D)** (*P <0.05).

In order to determine if autophagy following 3PO treatment serves as a survival mechanism, we assessed whether CQ, an agent that accumulates in lysosomes and interferes with autophagy, would promote or inhibit the cytotoxic effects of 3PO. Although exposure to 3PO alone caused a modest increase in late apoptotic (PI+/annexin-V+) HCT-116 cells, the addition of two non-toxic doses of CQ (15 or 30 μM) caused a dose-dependent increase in late apoptotic cells (Figure 6A, B). We also found that 30 μM CQ in combination with PFKFB3-specific siRNA transfection caused an increase in late apoptotic HCT-116 cells (Figure 6C, D). Two additional inhibitors of autophagy were then used in combination with 3PO in order to confirm that suppression of autophagy promotes 3PO-induced cell death. 3-methyladenine, an inhibitor of type III phosphatidylinositol 3-kinases which blocks the formation of autophagosomes, and Spautin-1, which inhibits autophagy by promoting increased proteasomal degradation of class III PI3 kinase complexes through inhibition of ubiquitin-specific peptidases USP10 and USP13, were both found to promote the pro-apoptotic effects of 10 μM 3PO (Figure 7A-D). In addition, we sought to examine the effect of siRNA-mediated suppression of autophagy on treatment with 3PO and chose to target ATG5, which is critical for the formation of the autophagosome [48]. We transfected HCT-116 cells with control siRNA or siRNA targeted to ATG5, followed by 24 hours of treatment with 10 μM 3PO and then examined the cells for apoptosis. We found that knockdown of ATG5 in the presence of 3PO caused a statistically significant increase in apoptotic cell death (PI+/annexin-V+ cells: control siRNA + vehicle, 5,100 ± 560; control siRNA + 10 μM 3PO, 6,800 ± 590 and ATG5 siRNA + 10 μM 3PO, 12,880 ± 630, *P* <0.05).

### CQ sensitizes Lewis Lung Carcinoma (LLC) cells to 3PO *in vitro* and *in vivo*

**Figure 8:**
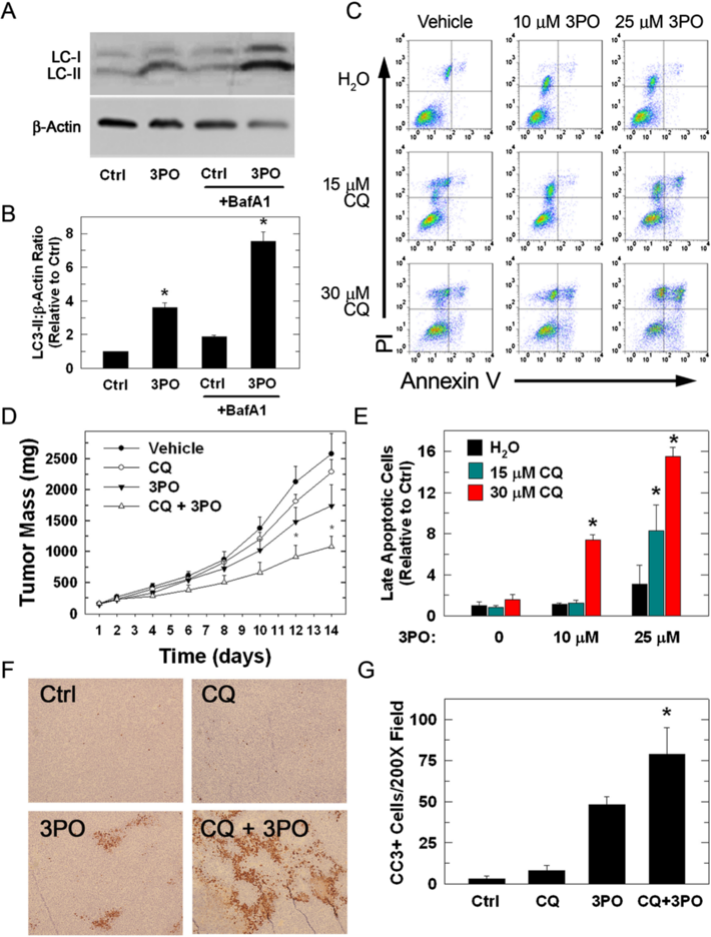
**Chloroquine increases the pro-apoptotic effects of 3PO against LLC cells and tumors *in vitro *and *in vivo*.** LLC cells were treated with 25 μM 3PO for 24 hours and LC3-II levels were measured using immunoblotting **(A)** and quantitative densitometry **(B)**. Levels expressed as mean fold change LC3-II/β-actin relative to control ± SD **(B)**. LLC cells were then treated with either vehicle or 10 or 25 μM 3PO ± either 15 or 30 μM CQ. After 24 hours of treatment, cells were stained with annexin-V and PI and measured using flow cytometry **(C)**. Cells staining positive for both annexin-V and PI were quantitated as the percentage of the total relative to control and data is presented as the mean ± SD from three experiments **(E)**. C57/BL6 mice were inoculated with 1x10^6^ LLC cells by subcutaneous flank injection. Mice were randomized into four treatment groups when tumors reached 150–200 mm^3^ and were treated by i.p. injections with either vehicle, 50 mg/kg CQ, 0.07 mg/g 3PO, or a combination of the two drugs. Tumor measurements taken over the course of treatment were used to calculate tumor mass. Data is presented as mean tumor mass ± SD **(D)**. Tumors were fixed, paraffin embedded, and stained with an antibody directed against cleaved caspase-3 (CC3) **(F)**. The number of cells staining positive for CC3 in five 200X fields were counted and the data is expressed as the mean ± SD from three counts **(G)** (*P <0.05).

We next transfected LLC cells with either control or PFKFB3-targeted siRNA followed by treatment with 0, 15 or 30 μM CQ and found that, similar to our findings in HCT-116 cells (Figure 6C, D), apoptosis was increased in the LLC cells treated with PFKFB3 siRNA and 30 μM CQ (PI+/annexin-V+ cells: control siRNA + vehicle, 4,200 ± 784; control siRNA + 30 μM CQ, 5,300 ± 890 and PFKFB3 siRNA + 30 μM CQ, 10,560 ± 1,630; *P* <0.05). We then exposed LLC cells to 25 μM 3PO and observed increased levels of LC3-II relative to control. This increase was further enhanced upon the addition of bafilomycin A1, which is consistent with increased autophagic flux (Figure 8A, B). Similar to the HCT-116 cells, LLC cell apoptosis caused by 3PO was increased by CQ (Figure 8C, E). Twelve-week-old female C57/BL6 mice then were injected subcutaneously with 1×10^6^ LLC cells and, when tumors reached 150–200 mm^3^, were randomized into four treatment groups (n = 6 per group): Group 1, vehicle (DMSO + PBS); Group 2, CQ (DMSO + 50 mg/kg CQ); Group 3, 3PO (0.07 mg/g 3PO + PBS); Group 4, CQ + 3PO (0.07 mg/g 3PO + 50 mg/kg CQ). Daily tumor measurements were obtained using micro-calipers and the experiment was concluded two weeks from the start of treatment. The tumor mass was significantly reduced in animals treated with both 3PO and CQ relative to either drug treatment alone (Figure 8D). Importantly, the mice did not exhibit any signs of increased toxicity including loss of body mass or gross pathological abnormalities of several organs. Excised tumors then were stained with an antibody recognizing cleaved caspase-3, a key protein in the execution phase of apoptosis. Tumors from animals treated with the combination of 3PO and chloroquine were noted to have an increased number of cleaved caspase-3 positive cells relative to tumors from animals treated with either drug alone (Figures 8F, G).

## Discussion

The metabolic stress caused by reduced glucose availability results in a number of cellular defense mechanisms critical to survive transitory periods of starvation. For example, energy-requiring processes are suppressed via the reduction of biosynthetic enzymes, inhibiting the activity of translational machinery and halting the cell cycle [49-51]. At the same time, catabolic processes, such as autophagy, are used to recycle intracellular components in order to provide metabolic substrates which can then be used to generate energy as well as to remove potentially harmful intracellular material such as damaged mitochondria [14,17,52-54].

In this study, we report that inhibition of PFKFB3 in HCT-116 cells increases the lipidated form of the autophagosomal membrane protein LC3 and decreases the cargo protein p62. LC3 is cleaved to LC3-I which liberates a C-terminal glycine that allows the conjugation to phosphatidylethanolamine whereupon the modified protein, called LC3-II, can target the autophagosomal membrane. Although counterintuitive, the heavier LC3-II migrates faster than LC3-I due to its hydrophobicity, and is seen as the lower band in Western blotting (Figures 2A, 4A, and 8A) [55,56]. Increased LC3-II can indicate either increased autophagic synthesis or reduced autophagic degradation. The addition of bafilomycin A1, an inhibitor of the vacuolar type H^+^-ATPase, allows for the determination of autophagic flux by inhibiting lysosomal acidification and blocking degradation of LC3-II [55,57,58]. The further increase in LC3-II protein that we observed in the presence of bafilomycin A1 after PFKFB3 inhibition indicated that PFKFB3 inhibition induced autophagy rather than blocked LC3-II degradation. Importantly, PFKFB3 inhibition also resulted in decreased p62 protein levels, an autophagy cargo receptor protein that contains an LC3-interacting region that targets it and its cargo to the autophagosome. In autophagy-competent cells, this cargo protein is degraded along with autophagosomal contents resulting in decreased total p62 [59]. Additionally, PFKFB3 inhibition resulted in cells with a higher volume of acidic compartments as measured using acridine orange staining, consistent with increased autophagy and, when visualized by electron microscopy, PFKFB3 inhibition also resulted in the appearance of autophagosomal structures. Taken together, these data are the first to demonstrate that PFKFB3 inhibition causes a compensatory increase in autophagy. Last, PFKFB3 inhibition resulted in decreased ATP, phospho-p70S6K, and phospho-S6 and an accumulation of ROS similar to that observed by glucose deprivation [36-39,60,61]. Each of these biochemical events can increase autophagy [41,42] and the increase in ROS mediated by 3PO was found to be essential for the induction of autophagy since N-acetylcysteine reversed the stimulation of autophagy caused by 3PO.

The identification of autophagy as a resistance mechanism utilized by tumor cells to avoid destruction and the induction of autophagy caused by PFKFB3 inhibition led us to postulate that the addition of autophagy inhibitors to a PFKFB3 small molecule antagonist would yield improved cytotoxic effects. In this report, we show that cell death following treatment with the PFKFB3 inhibitor 3PO was increased when combined with autophagy inhibitors CQ, 3-methyladenine or Spautin-1. Additionally, the combination of 3PO and CQ resulted in significantly smaller tumors relative to either drug treatment alone. Although our model system was different, the tumors from animals treated with CQ alone failed to show any difference in tumor size, contrasting with other published tumor studies [62,63]. Tumors that were removed from animals at the conclusion of the study were fixed and stained with a marker of apoptosis, cleaved caspase-3. This marker was increased in tumors excised from animals treated with the combination of CQ and 3PO relative to those from animals treated with either drug alone. The smaller tumor size and increased cleaved caspase-3 staining supports the idea that autophagy is serving as a protective mechanism following PFKFB3 inhibition and that the efficacy of PFKFB3 inhibitors as anti-cancer agents may be improved using autophagy inhibitors such as CQ.

## Conclusions

Harnessing the molecular information gained from studying cancer cells over the past century in order to determine the characteristics that distinguish them from normal cells is paramount to developing cancer-specific therapeutics. PFKFB3 inhibitors effectively and specifically target tumor cells *in vitro* and decrease tumor burden *in vivo*[10]. Importantly, a synthetic derivative of 3PO, termed PFK158, has undergone investigational new drug (IND)-enabling toxicology studies for the FDA and a Phase I clinical trial of its efficacy in advanced cancer patients is due to be initiated in early 2014 [64]. However, like so many chemotherapeutic agents, it is expected that resistance to these inhibitors will be encountered in clinical trials. Elucidating the specific resistance mechanisms triggered by targeted therapies allows for the selection of drug combinations that might work to combat such resistance with the hope of increasing efficacy. In this work, we show that autophagy is induced by PFKFB3 inhibition and that this induction is likely serving as a resistance mechanism given the observed increase in apoptosis *in vitro* and decrease in tumor growth *in vivo* mediated by pharmacologic inhibitors of autophagy. In conclusion, this study supports the further pre-clinical testing of rational combinations of PFKFB3 inhibitors with autophagy inhibitors for toxicity and efficacy in tumor-bearing animals.

## Abbreviations

CQ: Chloroquine; F2,6BP: Fructose-2,6-bisphosphate; F6P: Fructose-6-phosphate; G6P: Glucose-6-phosphate; HIF-1α: Hypoxia inducible factor 1 alpha; LC3-II: Microtubule-associated protein 1 light chain 3-II; LLC: Lewis lung carcinoma; PFKFB: 6-Phosphofructo-2-kinase/fructose-2,6-bisphosphatase; PFK-1: 6-Phosphofructo-1-kinase; PI: Propidium iodide; PTEN: Phosphatase and tensin homolog ROS, Reactive oxygen species; 2DG: 2-[1-^14^C]-deoxy-D-glucose; 3PO: (3-(3-pyridinyl)-1-(4-pyridinyl)-2-propen-1-one.

## Competing interests

BC, JC and ST are co-inventors on a related U.S. patent (#8,088,385) that is owned by the University of Louisville.

## Authors’ contributions

ACK conducted the majority of the studies in Figures 1, 2, 3, 4, 5, 6, 7 and 8 and prepared the initial draft of the manuscript. JO assisted ACK with *in vitro* apoptosis experiments and flow cytometry. JC and AC conducted the F2,6BP measurements and BC assisted with the autophagy characterizations. YIF assisted with the electron microscopy experiments. SRE assisted ACK with the interpretation of the autophagy experiments. JC directed the experimental design and interpretation of results presented in Figures 6, 7 and 8. ST conceived and directed the entire project with particular emphasis on Figures 1, 2, 3, 4 and 5. All authors assisted with the completion of the manuscript. All authors read and approved the final manuscript.

## Supplementary Material

Additional file 1: Figure S1 Transfection of HCT-116 cells with two separate PFKFB3-specific siRNA molecules induces autophagy. PFKFB3 and LC3-II protein levels were determined using Western blotting 48 hours after transfection with control (Ctrl) or two separate siRNA molecules directed against PFKFB3 (PFKFB3-1, PFKFB3-2) **(A)**. After 48 hours of transfection, HCT-116 cells were also stained with acridine orange, observed by fluorescent microscopy and collected by flow cytometry to measure the relative content of acidic compartments **(B,C)**. Data are presented as the mean ± SD from three experiments (*P* <0.05). Click here for file

Additional file 2: Figure S2 PFKFB3 inhibition with YN1 stimulates autophagy. HCT-116 cells were treated with either vehicle or 25 or 75 μM YN1 for 48 hours. F2,6BP concentration was measured **(A)**, viable cells were enumerated **(B)**, and LC3-II and p62 expression was measured by Western blot **(C)** and quantified by densitometry **(D)**. Data are presented as the mean ± SD from three experiments (*P* <0.05). Click here for file
