# Supplementary figures and images for: Inhibition of 6-phosphofructo-2-kinase (PFKFB3) induces autophagy as a survival mechanism
Source: Cancer Metab. 2014 Jan 23;2:2. doi: 10.1186/2049-3002-2-2 (PMC3913946; doi:10.1186/2049-3002-2-2)

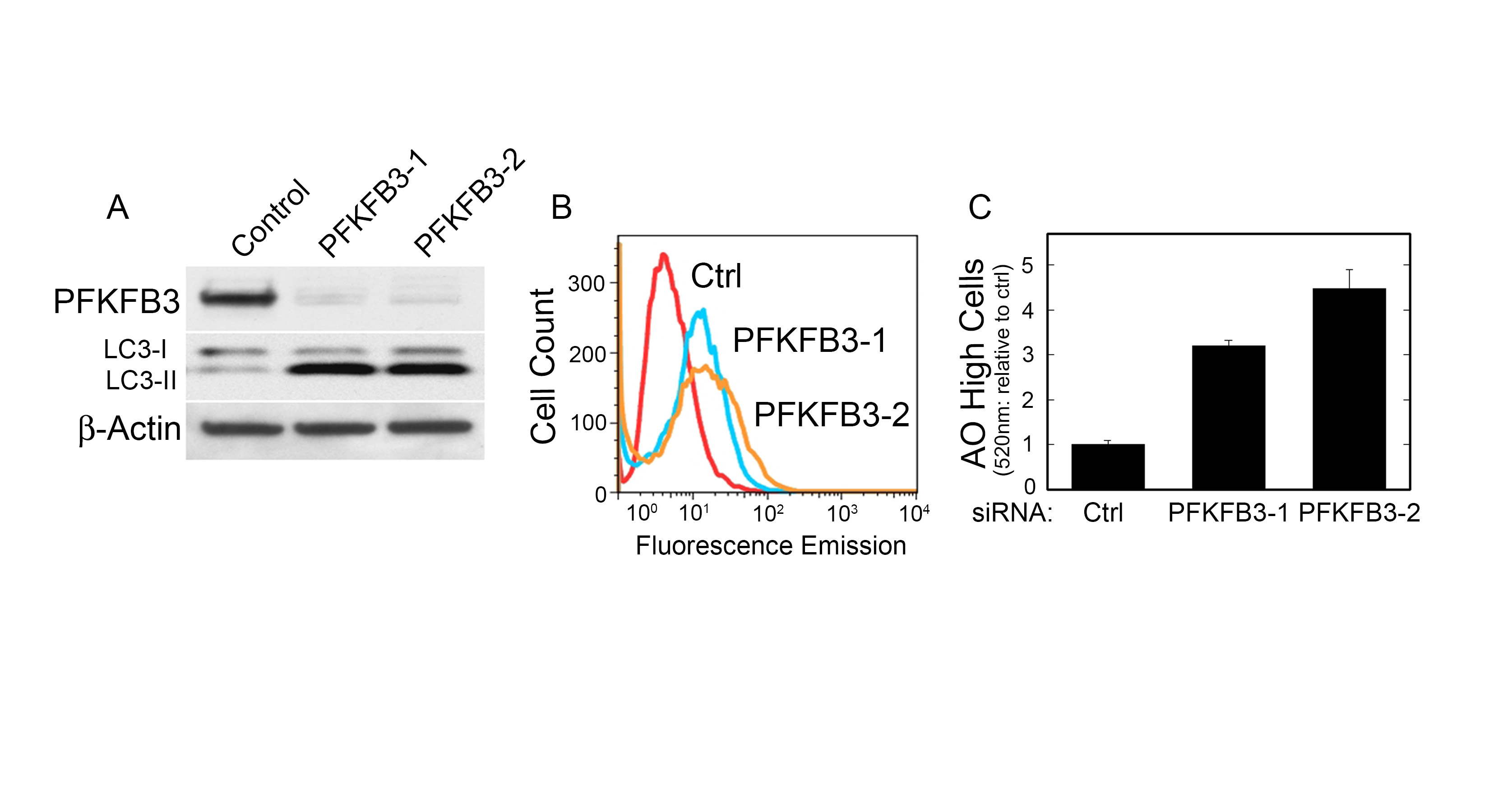

Supplement: Additional file 1: Figure S1 — Transfection of HCT-116 cells with two separate PFKFB3-specific siRNA molecules induces autophagy. PFKFB3 and LC3-II protein levels were determined using Western blotting 48 hours after transfection with control (Ctrl) or two separate siRNA molecules directed against PFKFB3 (PFKFB3-1, PFKFB3-2) (A). After 48 hours of transfection, HCT-116 cells were also stained with acridine orange, observed by fluorescent microscopy and collected by flow cytometry to measure the relative content of acidic compartments (B,C). Data are presented as the mean ± SD from three experiments (P <0.05). [file 2049-3002-2-2-S1.jpeg]

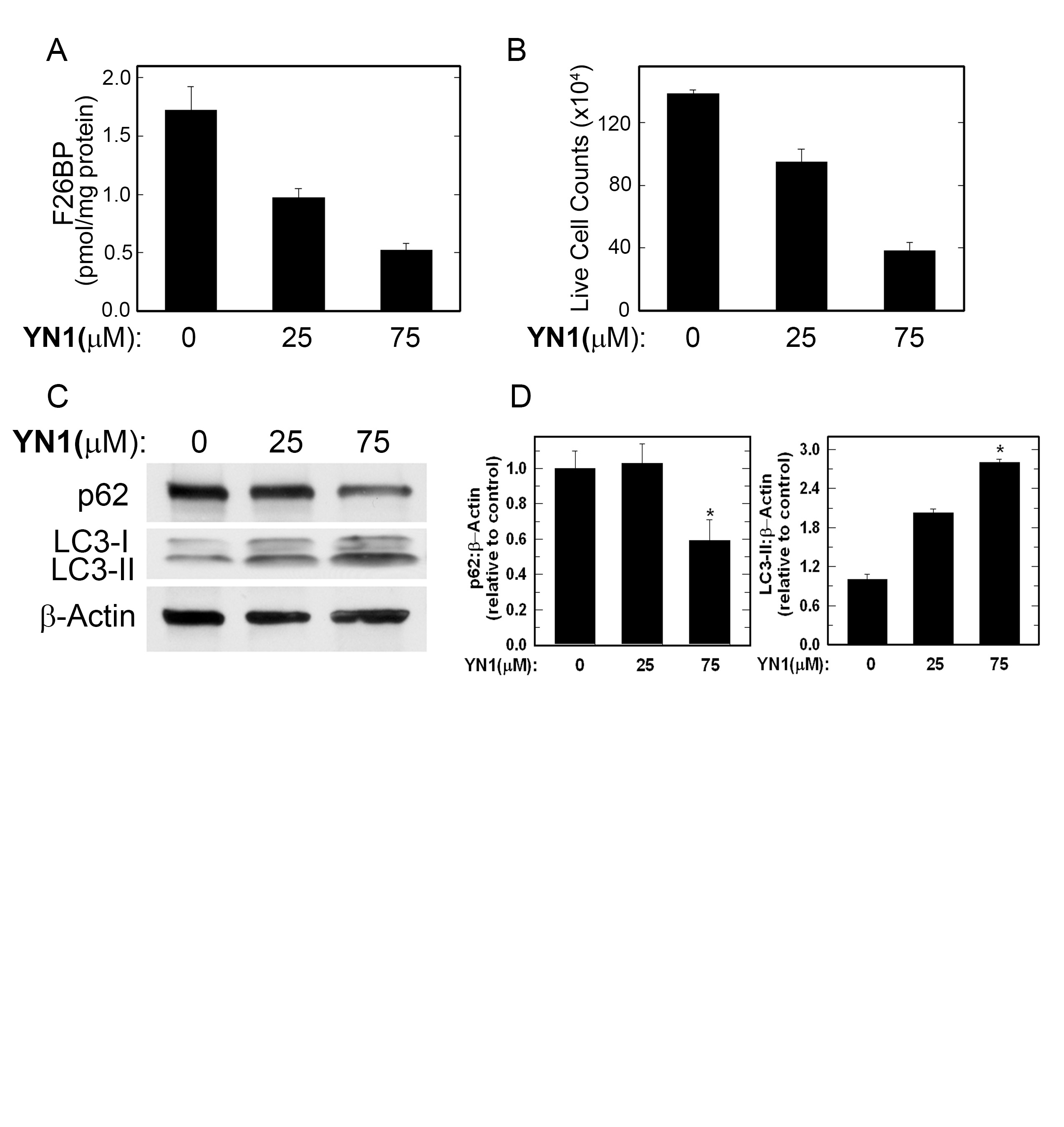

Supplement: Additional file 2: Figure S2 — PFKFB3 inhibition with YN1 stimulates autophagy. HCT-116 cells were treated with either vehicle or 25 or 75 μM YN1 for 48 hours. F2,6BP concentration was measured (A), viable cells were enumerated (B), and LC3-II and p62 expression was measured by Western blot (C) and quantified by densitometry (D). Data are presented as the mean ± SD from three experiments (P <0.05). [file 2049-3002-2-2-S2.jpeg]
